# Retraction Note: Enhanced tensile strength and thermal conductivity in copper diamond composites with B_4_C coating

**DOI:** 10.1038/s41598-022-25431-8

**Published:** 2022-12-06

**Authors:** Youhong Sun, Linkai He, Chi Zhang, Qingnan Meng, Baochang Liu, Ke Gao, Mao Wen, Weitao Zheng

**Affiliations:** 1 College of Construction Engineering, Jilin University, Changchun, 130026 People’s Republic of China; 2 Key Lab of Drilling and Exploitation Technology in Complex Conditions, Ministry of Land and Resources, Changchun, 130061 People’s Republic of China; 3 State Key Laboratory of Superhard Materials, Jilin University, Changchun, 130012 People’s Republic of China; 4 Department of Materials Science, Jilin University, Changchun, 130012 People’s Republic of China

Retraction of: *Scientific Reports* https://doi.org/10.1038/s41598-017-11142-y, published online 06 September 2017

Editors have retracted this Article.

Concerns were raised that some of the data in this paper appears to have been previously published in^1^ where it is described as representing different samples. Specifically, data in Figure 2 in this Article appears to be the same as data for sample D3 in Figure 1 in^1^; data in Figure 4a in this Article appears identical to data in Figure 2a in^1^ with the exception of the C-C peaks which appear to have been removed; data in Figure 4b in this Article appears to be identical to data in Figure 7a in^1^ with the exception of being shifted by approximately 6 eV. The Authors are not able to provide the original data due to the time that has passed since publication. The Editors no longer have confidence in the data reported in this Article.

Qingnan Meng agrees with the retraction. Editors were not able to establish the current contact details for other Authors.
